# Epilepsy Surgery in Children Under Three Years of Age

**DOI:** 10.15844/pedneurbriefs-33-3

**Published:** 2019-12-31

**Authors:** Hannah K. Weiss, S. Kathleen Bandt

**Affiliations:** Department of Neurological Surgery, Northwestern University, Chicago, IL

**Keywords:** Refractory Epilepsy, Development, Children, Focal Cortical Dysplasia, Hemispherotomy, Multi-Lobectomy

## Abstract

Researchers from the University Medical Centre Schleswig-Holstein, Epilepsy Centre Kork, University of Freiburg, University Children’s Hospital Heidelberg, Goethe University, and University Children’s Hospital Zürich conducted a study to evaluate seizure occurrence and cognitive development following epilepsy surgery in children under 3 years of age.

Researchers from the University Medical Centre Schleswig-Holstein, Epilepsy Centre Kork, University of Freiburg, University Children’s Hospital Heidelberg, Goethe University, and University Children’s Hospital Zürich conducted a study to evaluate seizure occurrence and cognitive development following epilepsy surgery in children under 3 years of age. Most of the patients underwent hemispherotomies (52%), followed by intralobar (35%) and multilobar (13%) resections. Etiologies included cortical malformations (71%), peri- or post-natal ischemic lesions (13%), benign tumors (8%), and tuberous sclerosis (8%). The patients were more likely to achieve seizure control following intralobar lesionectomy, rather than multilobar or hemispheric procedures. Seizure occurrence shortly after operation was predictive of long-term seizure recurrence. At the most recent follow-up, the majority of patients (60%) were found to be seizure-free and more than one-third (38%) had discontinued antiepileptic medications.

Presurgical cognitive development was found to have greater impairment with longer epilepsy duration and strongly corresponded with postsurgical cognitive development. The majority of patients (89%) had impaired postsurgical cognitive development. The authors proposed that early surgical intervention for epilepsy decreases the duration of the disease and improves long-term postsurgical cognitive development. [1]

COMMENTARY. Infants and young children have a high incidence and severity of epilepsy, and approximately one-third of them will develop drug-resistant epilepsy [2,3]. Intractable epilepsy in early childhood has been shown to be associated with long-term psychosocial and neurocognitive impairments [4,5]. In young children with intractable epilepsy, early diagnosis and longer duration of disease lead to greater cognitive disability [6]. The current study corroborates previously described associations between early surgical intervention and improved seizure and cognitive outcomes [7,8]. It demonstrates similar rates of seizure outcomes as found in previous studies [9] and is the first study to define an association between complete lesional resection and seizure freedom, specifically in children under the age of three. This study supports other studies identifying early postoperative seizures as a predictor of poor long-term seizure outcome [10]. The study suggests that epilepsy duration is a modifiable risk factor for developmental impairment and, therefore, early surgical intervention for children under the age of three suffering from drug-resistant epilepsy should be considered.

## Disclosures

The author(s) have declared that no competing interests exist.
